# Longitudinal serum proteomics identifies inflammatory and metabolic pathways in hypertensive nephrosclerosis progression

**DOI:** 10.1186/s12014-025-09537-5

**Published:** 2025-05-05

**Authors:** Ole Petter Nordbø, Øystein Eikrem, Philip A. Kalra, Hans-Peter Marti, Jessica Furriol

**Affiliations:** 1 Department of Clinical Medicine, University of Bergen, Bergen, Norway; 2 Department of Medicine, Haukeland University Hospital, Bergen, Norway; 3 Department of Medicine, Haugesund Hospital, Helse Fonna, Haugesund, Norway; 4 Department of Nephrology, Salford Royal Hospital, Northern Care Alliance NHS Foundation Trust, Salford, UK

## Abstract

**Background:**

Hypertensive nephrosclerosis (HN) is a major cause of end-stage renal disease; however, few longitudinal studies have employed serum proteomics to document its progression. This study aimed to identify potential circulating biomarkers indicative of disease progression in HN by performing serum proteomic analysis at two time points in patients with progressive and stable disease.

**Methods:**

Forty-one patients diagnosed with HN were recruited from the UK Salford Kidney Study, with serum samples collected at baseline and follow-up (1.5–10 years after baseline). Twenty-five patients experienced stable disease course, while 16 patients experienced progressive disease. Proteomics was performed via tandem mass tag labelling and liquid chromatography-tandem mass-spectrometry (LC–MS). Pathway analysis was performed on all significantly abundant proteins, as was network analysis of circulating proteins that are abundant in the kidney according to the Human Protein Atlas.

**Results:**

Pathway analysis revealed significant enrichment in pathways related to inflammation and infection, including complement and coagulation cascades, as well as metabolic processes in patients with disease progression. Marker abundance levels related to adhesion and the ECM were also altered in progresssive disease follow-up, compared to stable disease follow-up.

**Conclusion:**

The observed changes in inflammatory and adhesion-related pathways may offer valuable insights into the mechanisms driving HN progression and potential targets for intervention.

**Supplementary Information:**

The online version contains supplementary material available at 10.1186/s12014-025-09537-5.

## Introduction

Chronic kidney disease (CKD) is a global health concern. It leads to severe complications, increased mortality, cardiovascular disease, metabolic bone disease, and functional impairment, all of which contribute to significant healthcare utilisation [1–3]. Hypertension exacerbates the progression and impact of CKD, further increasing the burden of the disease [4, 5].

Recent advances in omics technologies have increased research to understand the molecular mechanisms of kidney diseases, including CKD [6]. Previous proteomics studies have identified biomarkers in urine [7, 8] and serum or plasma [9]. Specifically, urine proteomics has shown the potential to distinguish between slow and rapid disease progression [10].

Commonly used biomarkers like serum creatinine and urinary albumin excretion are non-specific, and changes often lag behind disease progression. Circulating proteins have emerged as potential biomarkers for predicting CKD progression. For instance, in 3235 participants in the Chronic Renal Insufficiency Cohort, 100 circulating proteins were identified to be associated with a 50% decline in estimated glomerular filtration rate (eGFR) or kidney failure over ten years, involving pathways such as bone morphogenetic proteins, ephrin signalling, and prothrombin activation [9]. In diabetic kidney disease, a kidney risk inflammatory signature of 17 proteins has been linked to a 10-year risk of end stage renal disease [11]. Other studies have identified proteins for classifying CKD progression via plasma [12] and urine [13] proteomics.

Despite these advances, serum proteomic studies on HN patients are lacking, and no known HN proteomics studies have incorporated follow-up sampling to evaluate the disease progression over time. In this study, we performed tandem mass tag (TMT) serum proteomics measurements at two time points in a cohort of HN patients categorised into progressive and stable CKD groups to identify circulating biomarkers related to disease progression and severity. LC–MS/MS with TMT labelling offers the advantage of multiplexing—simultaneous quantification of proteins from multiple samples in a single experiment. This increases throughput, reduces technical variability, and enhances the accuracy of comparative analyses between patient groups. Our approach addresses the need for longitudinal data to understand HN progression better and develop more effective predictive tools and therapeutic strategies.

## Methods

### Subjects and sample collection

Patients diagnosed with HN were recruited from the Salford Kidney Study (SKS), a non-dialysis CKD cohort containing samples and data from 3850 prospectively followed patients (as of 2024) [14]. The diagnosis of HN was determined through either clinical evaluation or histological confirmation. The cohort was divided into two groups: patients with slowly progressing (ΔeGFR < 1.6 ml/min/year) and rapidly progressing (ΔeGFR > 3 ml/min/year) HN, and serum was sampled at two time points during the study period for each group. Follow-up serum samples, including serum creatinine measurements, were collected from the same individuals between January 13, 2005, and March 22, 2016, with a follow-up period ranging from 1.5 to 10 years after the baseline collection during routine clinic visits. eGFR values were calculated via the CKD-EPI 2009 equation [15] using the egfr.ckdepi function from the kidney.epi R package (v. 1.2.0) [16].

### Protein depletion

Serum samples (10 µL each) were thawed on ice. Protein depletion was carried out via protein depletion mini spin columns (Cat. No. A36369; Thermo Scientific). Each depletion spin column was equilibrated to room temperature before use. Depleted samples were collected by centrifugation at 1000×*g* for 2 min.

### Protein denaturation

A buffer solution was prepared containing one protease inhibitor tablet (Cat. No. 04693132001; Roche) in 1 mL of molecular-grade water. To each dried sample, 40 µL of this buffer and 4 µL of 100 µM dithiothreitol in Milli-Q water were added. Each sample was vortexed, sonicated for 30 s, and incubated at 95 °C for 7 min. Protein alkylation was performed by adding 6 µL of freshly prepared 200 µM iodoacetamide in Milli-Q water for a total volume of 50 µL. The samples were incubated for 1 h at room temperature in the dark. The protein concentration was measured via the BCA protocol after denaturation. Reference samples for quality control were generated by pooling equal amounts of protein from the existing samples based on their measured protein concentrations.

### Solid-phase-enhanced sample preparation (SP3) and protein digestion

Initially, 300 µg of prepared SP3 beads (2 µL of a 150 µg/µL solution) were added to the protein mixture and homogenised by gentle pipetting. 100% ethanol was added to a final concentration of 70% to facilitate protein binding, followed by mixing. The binding mixture was incubated at room temperature for 7 min at 1000 rpm. The tube was placed in a magnetic rack until the beads migrated to the tube wall, and the unbound supernatant was discarded.

For bead rinsing, 180 µL of 80% ethanol SP3 rinse solution was added, and the beads were reconstituted and rinsed by pipette-mixing. The tube was placed on the magnetic rack to separate the beads, and the supernatant was removed without disturbing the beads. This rinse cycle was repeated twice more.

For digestion, 50 µL of digestion solution (100 mM ammonium bicarbonate/1 mM calcium chloride) containing a 1:25 enzyme-to-protein ratio of trypsin (Cat. No. V5111, Trypsin Porcine, Promega) was added to the beads. The mixture was sonicated for 30 s in a water bath to disaggregate the beads, followed by incubation for 18 h at 37 °C at 1000 rpm. After digestion, the tubes were centrifuged at 13,000 rpm at 24 °C for 3 min. The tube was placed on a magnetic rack to allow the beads to settle onto the tube wall, and the supernatant was transferred to a fresh tube. Then, 50 µL of 0.5 M sodium chloride solution was added, followed by pipette mixing. The tube was sonicated for 30 s in a water bath, centrifuged again at 13,000 rpm at 24 °C for 3 min, and then placed back on the magnetic rack to allow the beads to settle. The supernatant from this step was combined with the supernatant from the previous step.

### Sample desalting

The digested peptides were processed via OASIS C18 columns. Trifluoroacetic acid (TFA) (10%) was added to the trypsin‒sodium chloride mixture to a final concentration of 0.1% TFA. The volume was brought to 300 µL with an additional 0.1% TFA. The column was wetted with 500 µL of 70% acetonitrile (ACN) and 0.1% formic acid (FA) and conditioned twice with 500 µL of 0.1% TFA; each step involved centrifugation at 200×*g* for 1 min. The sample (300 µL) was loaded onto the column and centrifuged at 100×*g* for 3 min for binding. The samples were washed three times with 500 µL of 0.1% TFA, each followed by centrifugation. The peptides were eluted with two 100 µL aliquots of 70% ACN and 0.1% FA. After elution, the samples were dried via a freeze-vac and the dried peptide pellet was reconstituted in 20 µL of 0.5% FA and 2% ACN solution.

### TMT labelling

For TMT labelling, each sample was dissolved in 20 µL of 50 mM HEPES (pH 8), followed by mixing for 30 s on a thermoblock at 1500 rpm and ultrasonication for 30 s. The TMT ampoules were prepared by dissolving them in anhydrous acetonitrile to a concentration of 5 µg/µL. The mixture was vortexed and sonicated for 30 s every 2 min for 10 min. TMT labelling was performed at ten times the protein concentration, with two applications needed. In the first labelling step, 50 µg of TMT reagent was added to 10 µg of protein, mixed on a thermoblock at 1500 rpm, spun and incubated for 75 min. The second labelling step was repeated with 50 µg of TMT for the same amount of protein. After the second labelling, 5 µL of 10% hydroxylamine was added, and the mixture was incubated for 15 min. The samples were freeze-vac dried and redissolved in 500 µL of 0.1% TFA. The final desalting was conducted via a 10 mg OASIS plate (Cat. No. 186000128, Waters).

### Orbitrap eclipse mass-spectrometry

Following TMT labelling, LC/MS was performed. Briefly, high-field asymmetric waveform ion mobility spectrometry (FAIMS) was used to maximise the efficiency of data-dependent acquisition (DDA). Peptides were detected on an Orbitrap Eclipse Tribrid mass spectrometer (Cat. No. FSN04-10000, Thermo Scientific) using two compression voltages (CVs) of −50 and −70 V and “advance peak determination”. During each CV, the mass spectrometer was operated in DDA mode (data-dependent acquisition) to automatically switch between one full-scan MS and MS/MS acquisition. Instrument control was performed with an Orbitrap Eclipse Tune 3.5 and Xcalibur 4.5. The cycle time was maintained at 1.5 s/CV. MS spectra were acquired in the scan range of 400–1600 m/z with a resolution of R = 60,000 at m/z 200, an automatic gain control (AGC) target of 4e5 and a maximum injection time (IT) of 50 ms. The most intense eluting peptides with charge states of 2–7 were sequentially isolated to the standard target value (AGC, 1e5) and a maximum IT of 120 ms in the C-trap; the isolation width was maintained at 0.7 m/z (quadrupole isolation) before fragmentation during high-energy collision dissociation (HCD). Fragmentation was performed with a normalised collision energy (NCE) of 32%, and fragments were detected in the Orbitrap at a resolution of 50,000 at m/z 200, with the first mass fixed at m/z 110. One MS/MS spectrum of a precursor mass was allowed before dynamic exclusion for 30 s with “exclude isotopes” on. Lock‒mass internal calibration was not enabled. The spray and ion-source parameters were as follows. The ion spray voltage was 2000 V, there was no sheath or auxiliary gas flow, and the capillary temperature was 275 °C.

### Proteome data analysis

Raw mass spectrometric data were processed using Proteome Discoverer v.3.1 (Thermo Fisher). Peptide identification was performed against the human UniProt release 2022_03 database using the Sequest HT search engine. Peptide identifications were filtered, applying a minimum peptide confidence level of “Medium” or “High” based on the false discovery rate (FDR); peptides with “Low” confidence were excluded. Proteins accepted for further analysis had at least one unique peptide and an overall confidence of “Medium” or “High”. Normalisation was performed using the total peptide amount normalisation method (default in Proteome Discoverer), which adjusts for variations in total signal intensity within each batch. Batch effects across different experimental runs were further assessed and corrected during downstream analysis in R (v4.4.1).

To handle missing data and potential batch effects, peptides with more than 85% missing values across all samples or missing values in more than three of the eight experimental batches were removed. Potential contaminants such as keratins and bovine serum peptides, along with the top 14 most abundant serum and red blood cell proteins (Table S1), were excluded before differential expression analysis.

We calculated the percentage of missing values for each protein in each batch and assessed missingness within each condition across batches. Proteins with less than 50% missing data in at least one clinical subgroup—Non-progressor baseline, Progressor baseline, Non-progressor repeat, or Progressor repeat—were selected for imputation. The data were stratified into these subgroups, and missing peptide values were imputed using random forest regression via the missForest (v1.5.0) package. Proteins with high missingness in reference samples (>50%) were excluded. In cases where insufficient data were available within a subgroup to perform regression imputation, mean imputation was applied instead.

Differential expression analysis was performed using the limma (v3.60.3) package in R, employing a paired linear model to account for repeated measures within each patient, with age included as an additional covariate. Specifically, a “Pair” factor identified samples from the same individual in comparisons of progressor baseline (PBL) vs. progressor follow-up (PF), PBL vs. stable baseline (SBL), PF vs. stable follow-up (SF), and SBL vs. SF.Although normalisation was performed at the peptide level in Proteome Discoverer, additional batch correction was applied at the protein level using the removeBatchEffect function in limma to further reduce unwanted technical variation due to differences in batches. To control for multiple hypothesis testing, we utilised the Benjamini–Hochberg procedure for FDR correction to adjust p-values, with a threshold of FDR ≤ 0.05 for significance. Heatmaps and uniform manifold approximation and projection (UMAP) analysis detailing the handling of batch effects is shown in Fig S1. UMAP is sensitive in capturing subtle, non-linear variations within the data, and might be a better chosise for inspecting potetnial batch effects compared with PCA. Heatmaps were generated via the ComplexHeatmap package (v 2.20.0), and UMAP plots were generated via the umap package (v 0.2.10.0).

Principal component analysis (PCA) was conducted on the abundance data via the prcomp function using scaling, with visualisation via the factoextra (v 1.0.7) package. Pathway analysis was performed using the pathfindR (v2.3.1) package with default arguments. This algorithm first identifies “active subnetworks” of interacting proteins that predominantly contain significantly altered proteins, then performs an over-representation analysis on the proteins in each subnetwork. Differentially expressed proteins (DEPs) were identified on the basis of the log_2_fold change (log_2_FC) and *p*-values < 0.05. Enriched terms were clustered in pathfindR, which utilises pairwise kappa statistics between enriched terms, performs hierarchical clustering, and determines the optimal number of clusters. pathfindR then reports an aggregated list of the enriched pathways and their associated proteins. The term–protein relationships were visualised using the term_gene_graph function. In the enrichment analyses, we applied FDR correction (FDR < 0.05), reporting only the significant pathways after correction.

Protein abundance in the kidney was checked via the Human Protein Atlas (HPA) histology database for normal tissue via the hpar (v 1.46.0) and HPAanalyze (v 1.22.0) packages. The HPA category “predicted to be secreted” was used to annotate potentially secreted proteins. Transcription factor activity was determined by matching proteomics data with known transcription factor related gene sets via the msigdbr (v 7.5.1) package. The gene set IDs for determining transcription factor activity included M18775, M26112, M29447, M26863, M4864, M29507, M14355, and M18797. Protein‒protein interaction (PPI) partners were identified via the STRINGdb (v 2.16.4) package [17] by mapping DEPs to known interactions from the STRING database. Only interactions with a confidence score of 0.4 or higher (medium confidence threshold) were retained to reduce false positives. The graphs were generated via the igraph package (v 2.0.3) using the Fruchterman–Reingold algorithm, which arranges network nodes to optimise clarity by evenly spacing them and reducing overlap among connections. Lowly connected nodes (those with fewer than three interactions or isolated nodes) were removed from the network to focus on proteins with more substantial connectivity.

## Results

### Baseline characteristics

The baseline clinical characteristics of the progressive and stable patients are shown in Table 1. The study included 41 participants, divided into stable (N = 25) and progressor (N = 16) groups with baseline and follow-up proteomic measurements. The gender distribution was 63% male and 37% female overall, with 69% males in the progressor group and 60% in the stable group. At baseline, the mean age was 69 years (SD ± 11), with progressors averaging 71 years and the stable group 67 years. The mean eGFR was lower in the progressor group (29 mL/min/1.73 m^2^) than in the stable group (36 mL/min/1.73 m^2^). The prevalence of diabetes was 17% across both groups. The mean body mass index (BMI) was 29 kg/m^2^ (SD ± 5.3), with 10% of the data missing. Blood pressure readings showed a mean systolic blood pressure (BP) of 140 mmHg (SD ± 22) and a diastolic BP of 74 mmHg (SD ± 14) in the stable group and 150 mmHg (SD ± 16) and 71 mmHg (SD ± 8.6) in the progressor group. A history of myocardial infarction was present in 17% of the participants, with a higher prevalence in the stable group (24%). Overall survival was 63%, with 50% overall survival in progressors and 72% overall survival in stable participants. Fifteen percent of the patients required renal replacement therapy, with 25% in the progressor group and 8% in the stable group. *p*-values were calculated using the *t*-test (for continuous variables) and chisq.test (for categorical variables) functions in R, respectively.

**Table 1:** Baseline characteristics of the study population

	Stable (N = 25)	Rapid (N = 16)	*p*-value
Gender			
Male	15 (60%)	11 (69%)	0.814
Female	10 (40%)	5 (31%)	
Age (years)			
Mean (SD)	67 (±11)	71 (±12)	0.248
eGFR (mL/min/1.73m^2^)			
Mean (SD)	36 (±19)	29 (±8.7)	0.137
Diabetes			
Yes	5 (20%)	2 (12%)	0.844
No	20 (80%)	14 (88%)	
BMI (kg/m^2^)			
Mean (SD)	29 (±5.4)	28 (±4.6)	0.394
Systolic BP (mmHg)			
Mean (SD)	140 (±22)	150 (±16)	0.244
Diastolic BP (mmHg)			
Mean (SD)	74 (±14)	71 (±8.6)	0.48
Myocardial infarction			
No	19 (76%)	15 (94%)	0.295
Yes	6 (24%)	1 (6%)	
Alive			
Yes	18 (72%)	8 (50%)	0.274
No	7 (28%)	8 (50%)	
RRT			
Yes	2 (8%)	4 (25%)	0.294
No	23 (92%)	12 (75%)	

*eGFR* estimated glomerular filtration rate, *BMI* body mass index

**Fig. 1:**
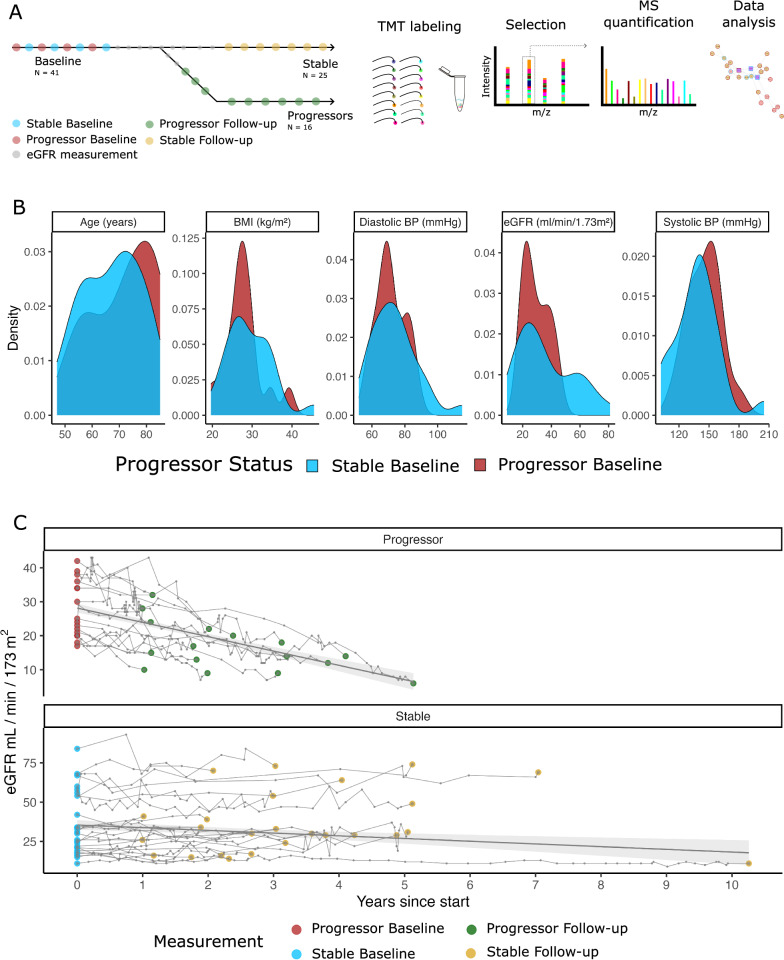
Study overview and baseline characteristics of kidney function and clinical data. **A** The study included 41 patients divided into two groups: stable (n = 25) and progressive (n = 16). Serum samples were collected from each patient twice during follow-up (baseline and follow-up) for proteome measurements. **B** Density plots showing baseline clinical characteristics for stable and progressive patients, including age (years), BMI (kg/m^2^), diastolic and systolic blood pressure (mmHg), and eGFR (ml/min/1.73 m^2^). **C** eGFR curves illustrating changes over time between samples for both groups. The eGFR was recorded at semiregular intervals. Colors: *red* for PBL, *green* for PF, *blue* for SBL, and *yellow* for SF. *BMI* body mass index, *eGFR* estimated glomerular filtration rate, *BP* blood pressure

We analysed 25 baseline and follow-up samples from the stable group (eGFR loss of −1.6 mL/min/1.73 m^2^/year) and 16 baseline and follow-up samples from the progressor group (eGFR loss of −4.5 mL/min/1.73 m^2^/year) (Fig. 1a). The clinical data distribution at baseline is shown in Fig. 1b, indicating closely matched cohorts. eGFR curves for the stable and progressive groups were calculated based on creatinine levels (Fig. 1c). The eGFR curves start and end with the first and last samples used in the proteomics experiment. The progressive group presented a more significant decrease in the eGFR (Fig. 1d). The time intervals between measurements ranged from 1 to 10 years after the baseline measurement. Complete eGFR curves for all available data before and after proteomics sampling are shown in Fig S2.

### Sample overview and differentially expressed proteins

**Fig. 2:**
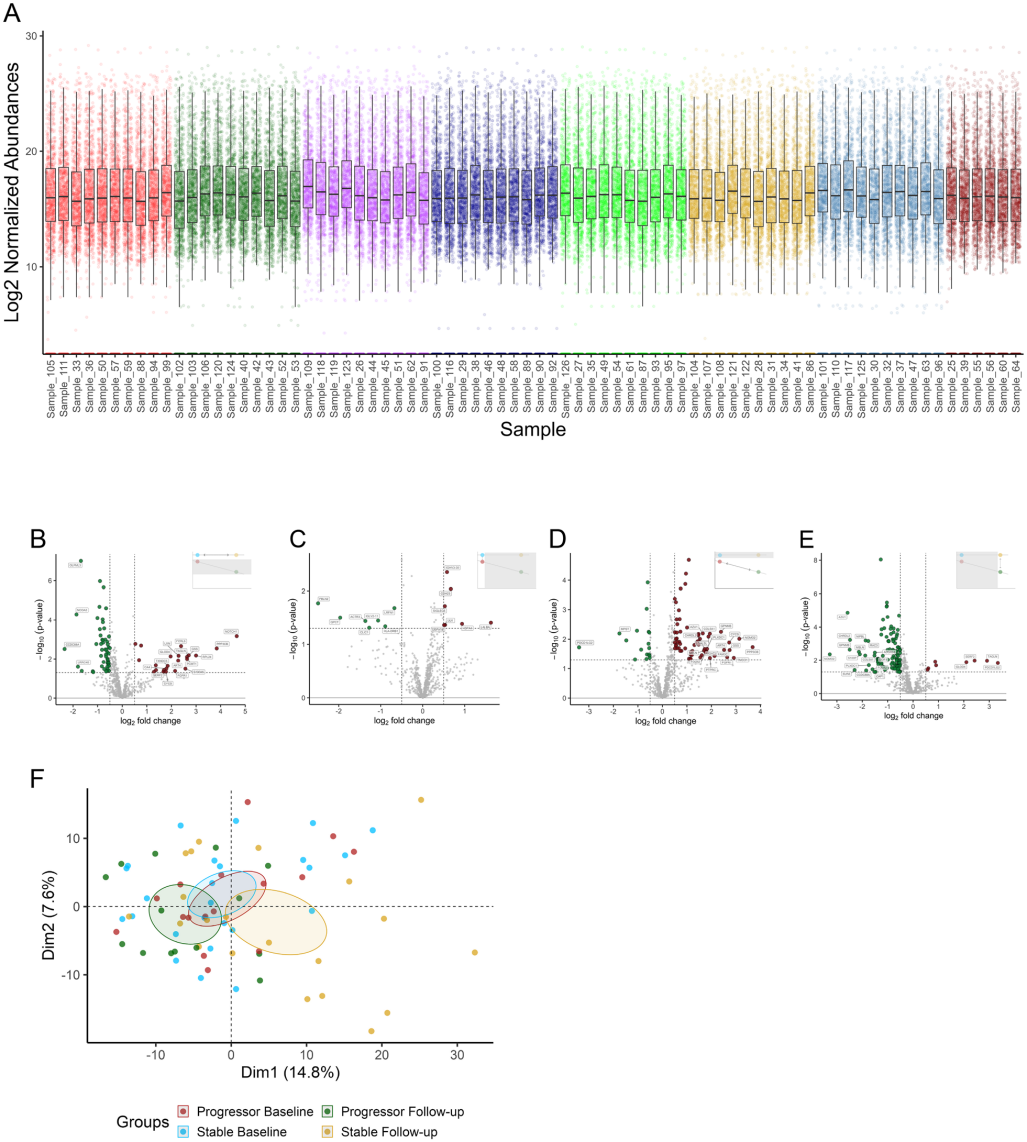
Sample overview and differentially expressed proteins. **A** log_2_ normalised peptide abundances for each sample, colored by batch (1–8) to visualise potential batch effects and the overall distribution of peptide measurements. **B** Volcano plots of DEPs with log_2_ FC and −log10 *p* values in stable baseline vs. stable follow-up, **C** progressor baseline vs. stable baseline, **D** progressor baseline vs. progressor follow-up, and **E** progressor follow-up vs. stable follow-up. **F** PCA of significantly expressed proteins (*p* < 0.05) in each group. colors: *red* progressor baseline, *green* progressor follow-up, *purple* stable baseline, *yellow* stable follow-up

The log_2_ normalised peptide abundances across samples are shown in Fig. 2a. The median log_2_ values were consistent across batches, with only slight variations. After contaminants and proteins with excessive missing data were filtered out, 721 proteins were included in the analysis. Volcano plots display the DEPs for each comparison: 75 DEPs in SBL vs. SF (Fig. 2b), 14 DEPs in PBL vs. SBL (Fig. 2c), 79 DEPs in PBL vs. PF (Fig. 2d), and 121 DEPs in PF vs. SF (Fig. 2e). The PCA plot (Fig. 2f) shows a clear overlap between the SBL and PBL groups, indicating minimal differences in global protein abundance, whereas the SF and PF groups show a slight separation. PCA plots annotated with other clinical parameters are shown in Fig. S3. The output data proteome discoverer is given in Table S2, and output for the differential expression analysis in Table S3.

### Protein enrichment analysis

To identify pathways enriched for proteins in more severe disease states and dysregulated pathways under baseline conditions, we performed protein enrichment analysis via the pathfindR package. DEPs with *p* values <0.05 and absolute log_2_ FC > 0.5 were used as inputs in the enrichment analysis. For the pathway cluster analysis, pathways with less than 4 enriched proteins were excluded, in addition to redundant pathways related to cancer or other terms. In the cluster network analysis, no cutoff was used to provide a full overview of processes involved. Unfiltered pathway enrichment data is given in Table S4. Too few DEPs to generate a meaningful pathway analysis were identified between the baseline comparisons.

**Fig. 3:**
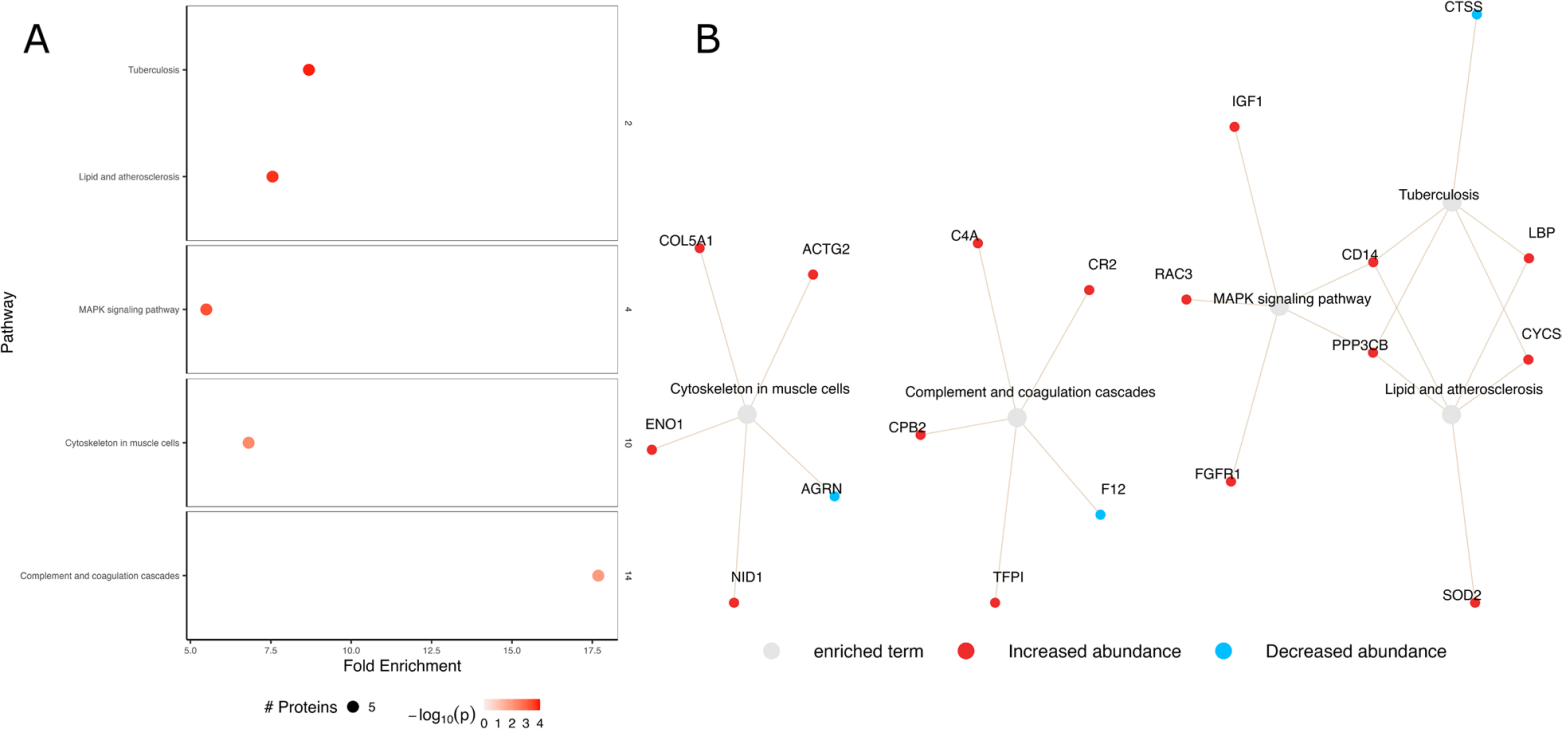
Pathway enrichment analysis of progressive baseline (PBL) vs. progressive follow-up (PF) measurements. **A** Selected enriched pathways and their respective clusters for PBL vs. PF. **B** Network analysis of genes associated with the clustered terms from the PBL vs. PF comparison in **A**. Network analysis: *grey nodes* represent pathways, *red/blue nodes* represent proteins, colors: *red*—increased abundance, *blue*—decreased abundance. The −log_10_ *p*-value is shown by increasing *red intensity*. The size of each node corresponds to the number of enriched proteins in the pathway

**Fig. 4:**
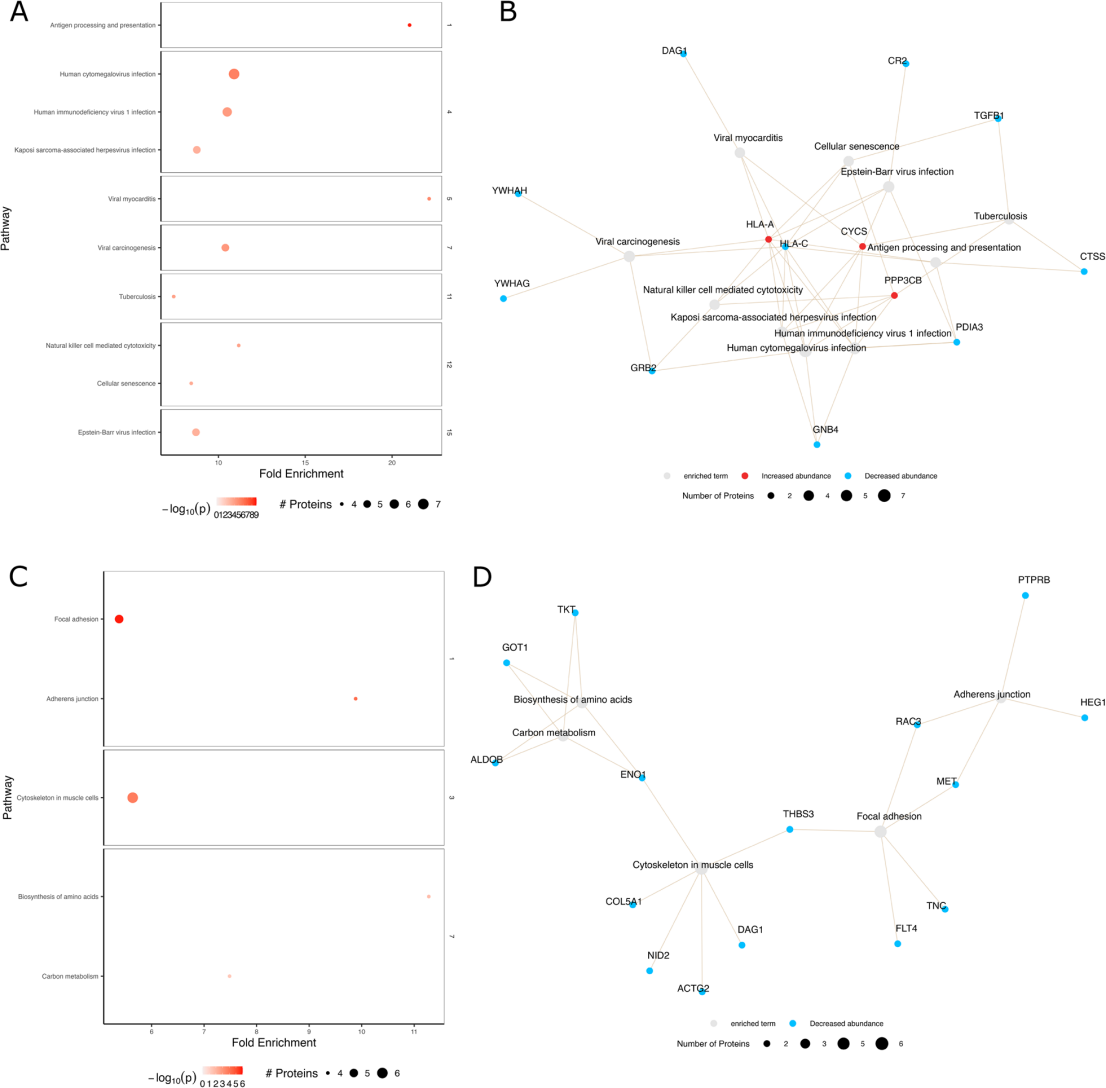
Pathway enrichment analysis of stable baseline (SBL) vs. stable follow-up (SF) and progressor follow-up (PF) vs. stable follow-up (SF) measurements. **A** Selected enriched pathways and their respective clusters for SBL vs. SF, with clusters related to inflammation and infection, metabolism, N-glycan biosynthesis and relaxin signalling. **B** Network analysis of genes associated with the clustered terms from the SBL vs. SF comparison in **A**. **C** Clustered terms for PF vs. SF, resulting in 4 clusters related to ECM/cytosceleton, infection and inflammation, adhesion and amino acid/carbohydrate metabolism. **D** Network analysis of genes linked to the clustered terms from the PF vs. SF comparison in **C**. Network analysis: *grey nodes* represent pathways, *red/blue nodes* represent proteins, colors: *red*—increased abundance, *blue*—decreased abundance. The −log_10_ *p*-value is shown by increasing *red intensity*. The size of each node corresponds to the number of enriched proteins in the pathway

In the PBL vs. PF comparison (Fig. 3a, b), pathways related to inflammation (including complement) and atherosclerosis were enriched, alongside dysregulation of MAPK signaling and cytoskeleton regulation. In the SBL vs. SF groups (Fig. 4a, b), most proteins exhibited decreased abundance, with enriched pathways involving inflammation, infection, and cellular senescence. When comparing PF to SF (Fig. 4c, d), decreased protein abundance was observed in the PF group, and the enriched pathways were associated with adherence junction, focal adhesion, amino acid synthesis, and carbon metabolism.

### Network analysis

The serum proteome includes proteins from various tissues, including the kidney, as well as endogenous blood proteins. To focus our analysis on kidney-related proteins, we identified proteins abundant in the kidney via HPA v24, which we used as basis for the analysis. To infer which markers might hold potential as important biological agents with several interaction partners, we generated protein–protein interaction (PPI) graphs for each comparison, excluding the PBL vs. SBL comparison due to an insufficient number of DEPs. Network visualisation was performed using the igraph package, employing the Fruchterman–Reingold algorithm to optimize node distribution and minimise overlap. Respective density plots were generated to highlight abundance patterns in the respective renal compartments, including proximal tubules (cell bodies and microvilli), distal tubules, cells in tubules, collecting ducts, cells in the glomeruli, and Bowman’s capsules.

**Fig. 5:**
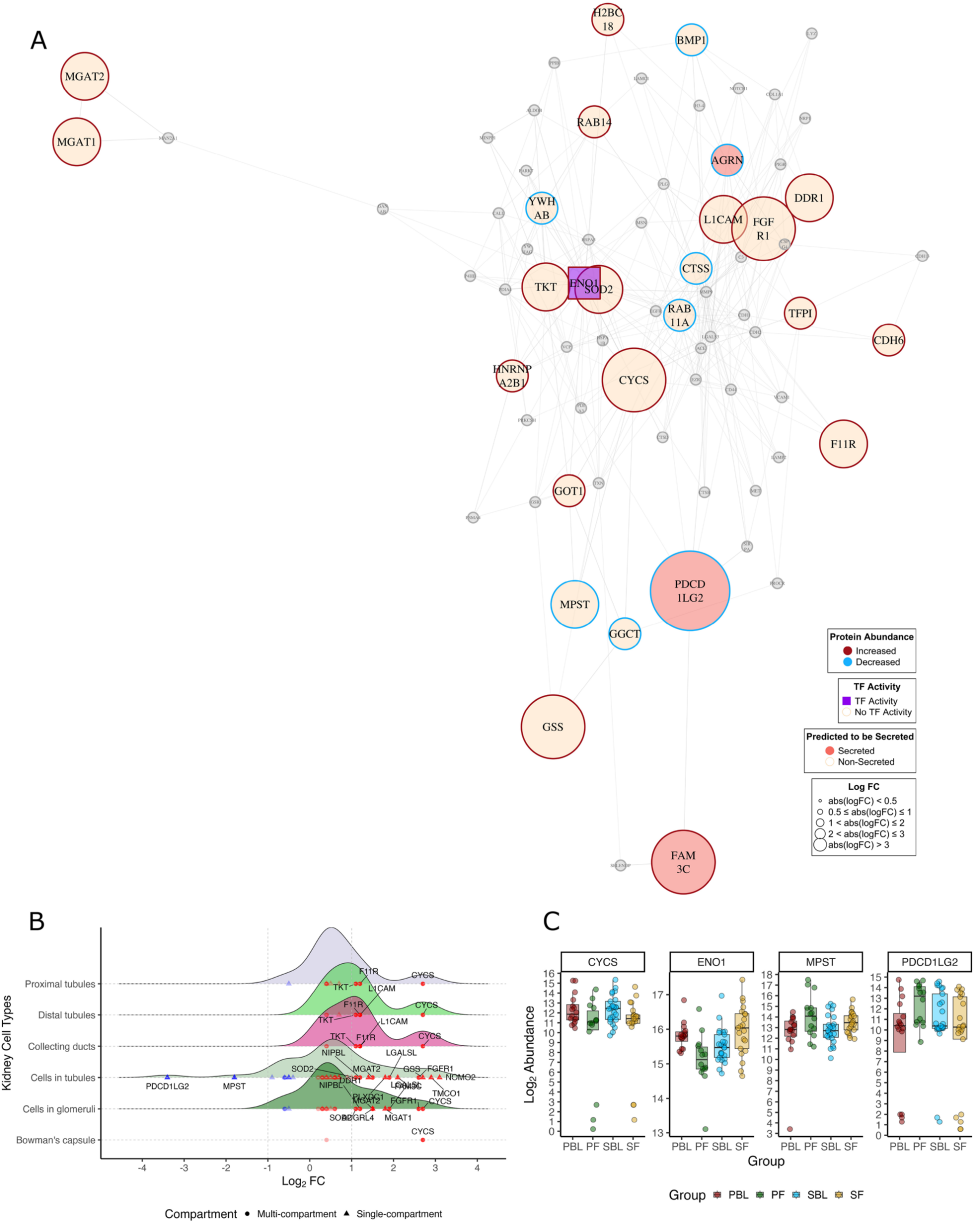
Network and expression profiles of kidney proteins for progressor baseline (PBL) vs. progressor follow-up (PF). **A** Protein–protein interactions of significantly abundant kidney proteins in PBL vs. PF (n = 72 nodes, n absolute logFC > 0.5 = 26). Nodes represent individual proteins, and edges represent protein–protein interactions. Proteins with increased abundance are displayed with *red rims*, whereas *blue rims* represent decreased abundance. Proteins predicted to be secreted (n = 3) are highlighted as *pink nodes*, whereas proteins with transcription factor activity (n = 1) are represented as *purple squares*. Significantly expressed kidney proteins (*p* < 0.05) with absolute log_2_ FC < 0.5 are represented as *grey nodes*. The graph was generated via the Fruchterman-Reingold algorithm. Nodes with fewer than three interactions, or isolated nodes were removed from the network. **B** Density plots depicting significant proteins (*p*-value < 0.05) expressed in renal compartments: proximal tubules, distal tubules, collecting ducts, cells in tubules, cells in glomeruli, and Bowman’s capsule. The distribution of log_2_ FC expression values for DEPs within the respective compartments is shown. Proteins expressed in multiple renal compartments are depicted as *circles*, whereas single-compartment proteins are depicted as *triangles*. **C** Boxplots showing the log_2_ abundance levels for selected markers CYSC, ENO1, MPST and PDCD1LG2

The protein–protein interaction network displayed connected nodes in the PBL/PF graph (Fig. 5a). The highly connected nodes included enolase 1 (*ENO1*) and cytochrome c (*CYCS*), with three DEPs predicted to be secreted. The majority of dysregulated proteins originated from the tubules or glomeruli (Fig. 5b), including increased abundance of *NOMO2* and transmembrane and coiled-coil domains 1 (*TMCO1*), and decreased abundance of programmed cell death 1 ligand 2 (*PDCD1LG2*) and 3-mercapto pyruvate sulfurtransferase (*MPST*) (Fig. 5c).

**Fig. 6:**
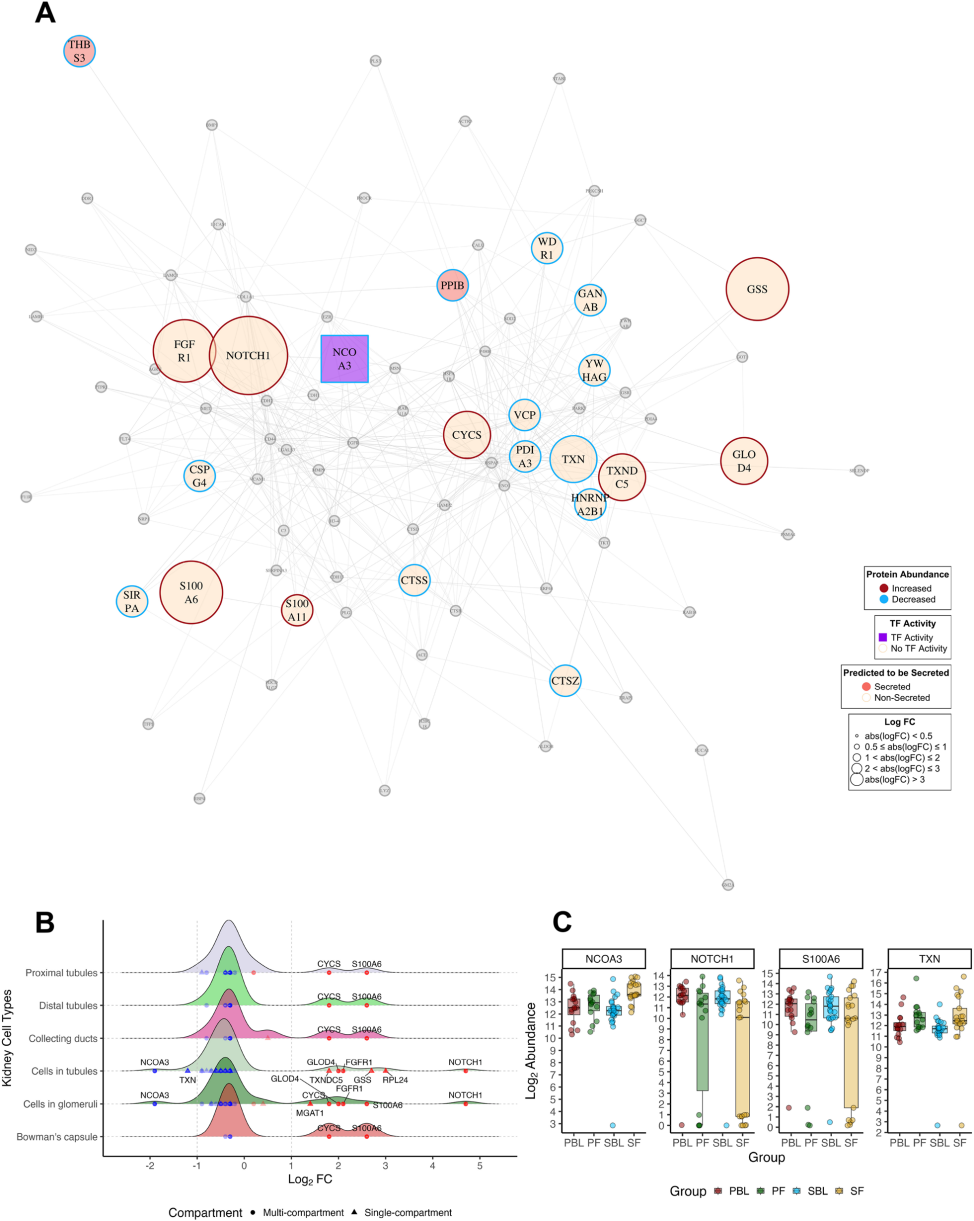
Network and Expression Profiles of Significant Kidney Proteins for Stable Baseline vs. Stable Follow-up. **A** Protein–protein interactions of significantly abundant kidney proteins in SBL vs. SF (n = 85 nodes, n absolute logFC > 0.5 = 22). Nodes represent individual proteins, and edges represent protein–protein interactions. Proteins with increased abundance are displayed with red rims, whereas *blue rims* represent decreased abundance. Proteins predicted to be secreted (n = 2) are highlighted as *pink nodes* whereas proteins with transcription factor activity (n = 1) are represented as *purple squares*. Significantly expressed kidney proteins (*p* < 0.05) with absolute log_2_ FC < 0.5 are represented as *grey nodes*. The graph was generated via the Fruchterman-Reingold algorithm. Nodes with fewer than three interactions, or isolated nodes were removed from the network. **B** Density plots depicting significant proteins (*p*-value < 0.05) expressed in renal compartments: proximal tubules, distal tubules, collecting ducts, cells in tubules, cells in glomeruli, and Bowman’s capsule. The distribution of log_2_ FC expression values for DEPs within the respective compartments is shown. Proteins expressed in multiple renal compartments are depicted as *circles*, whereas single-compartment proteins are depicted as *triangles*. **C** Boxplots showing the log_2_ abundance levels for selected markers NCOA3, NOTCH1, S100A6 and TXN

In the SBL vs. SF graph, nodes are clustered according to their abundance levels (Fig. 6a). Two proteins were predicted to be secreted, as well as one transcription factor, all with decreased abundance under SBL conditions. Some notable highly connected nodes included neurogenic locus notch homologue protein 1 (*NOTCH1*, in glomerular and tubular cells), S100 calcium-binding protein A6 (*S100A6*, most compartments except tubular cells) was also observed. A decreased abundance of nuclear receptor coactivator 3 (*NCOA3*, Log_2_ FC −2, tubules, and glomeruli) and thioredoxin (*TXN*, Log_2_ FC −1.2, tubules only) was detected (Fig. 6b, c).

**Fig. 7:**
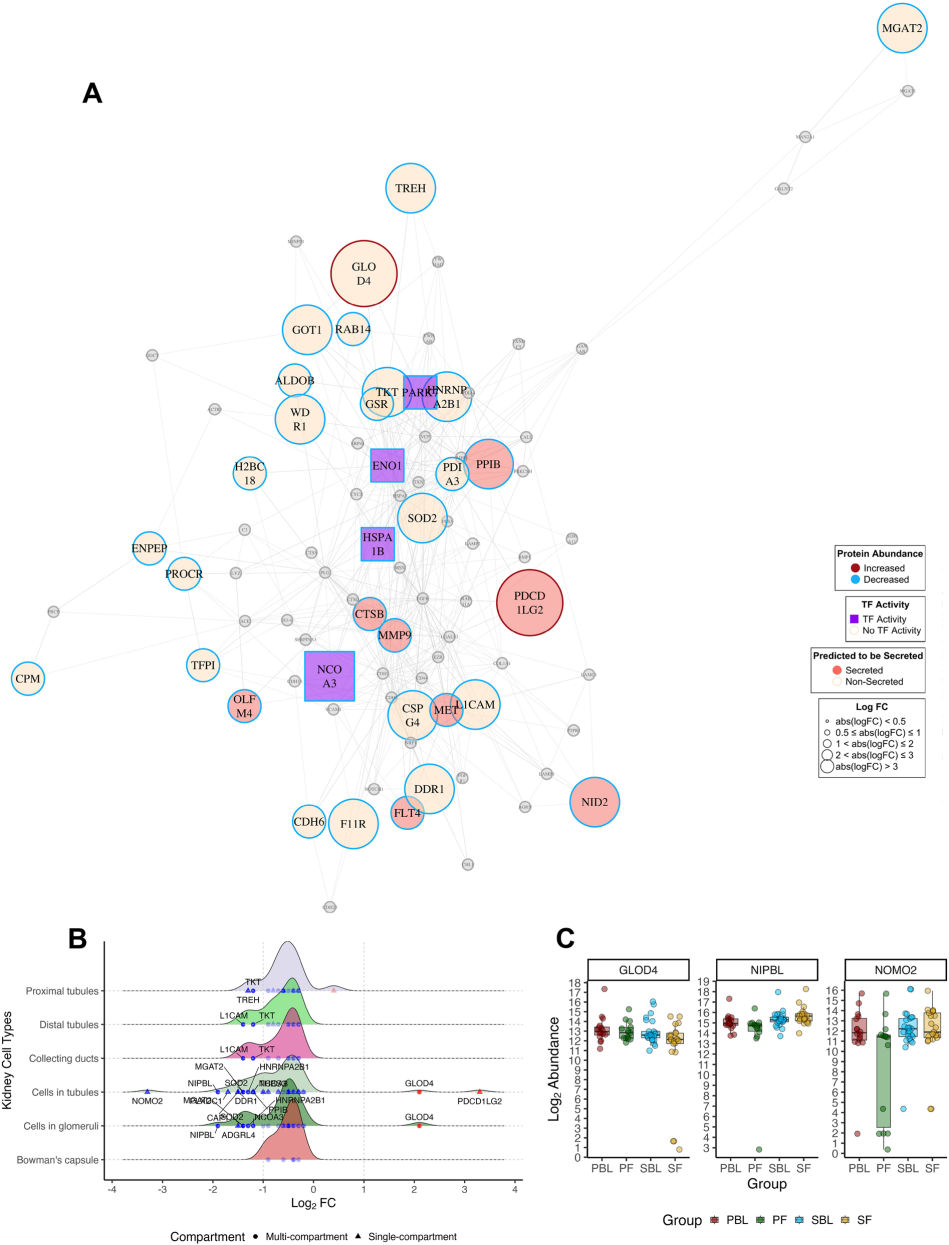
Network and expression profiles of significant kidney proteins for progressive follow-up (PF) vs. stable follow-up (SF). **A** Protein–protein interactions of significantly abundant kidney proteins in PF vs. SF (n = 86 nodes, n absolute logFC > 0.5 = 34). Nodes represent individual proteins, and edges represent protein–protein interactions. Proteins with increased abundance are displayed with *red rims*, whereas blue rims represent decreased abundance. Proteins predicted to be secreted (n = 8) are highlighted as *pink nodes*, whereas proteins with transcription factor activity (n = 4) are represented as *purple squares*. Significantly expressed kidney proteins (*p* values < 0.05) with absolute log_2_ FC < 0.5 are represented as grey nodes. The graph was generated via the Fruchterman-Reingold algorithm. Nodes with fewer than three interactions, or isolated nodes were removed from the network. **B** Density plots depicting significant proteins (*p*-value < 0.05) expressed in renal compartments: proximal tubules (microvilli and cell body), distal tubules, collecting ducts, cells in tubules, cells in glomeruli, and Bowman’s capsule. The distribution of log_2_ FC (log_2_FC) expression values for DEPs within the respective compartments is shown. Proteins expressed in multiple renal compartments are depicted as *circles*, whereas single-compartment proteins are depicted as *triangles*. **C** Boxplots showing the log_2_ abundance levels for selected markers GLOD4, NIPBL, and NOMO2

Network analysis of the PF/SF group showed 8 kidney proteins predicted to be secreted and 4 transcription factors (Fig. 7a). Assessment of renal compartment abundance levels showed a broad range of DEPs from all compartments, except for Bowman’s capsule, which had no protein with absolute Log_2_ FC > 1. Most renal proteins in the PF group, except the glyoxalase domain containing 4 (*GLOD4*) and *PDCD1LG2*, presented decreased abundance. *NOMO2* (Log_2_ FC −3.2) and Nipped-B-like protein (*NIPBL*, Log_2_ FC −2) were the most decreased renal proteins in PF, whereas *PDCD1LG2* and *GLOD4* had the most increased abundances (Fig. 7b, c).

## Discussion

This study presents a comprehensive proteomics analysis of HN patients. We divided the patients into groups based on their disease progression status (Stable and Progressor) at baseline and follow-up. By tracking the same patients over time, we identified specific protein markers associated with the progression of CKD. Baseline measurements enabled comparisons to identify prognostic and disease-associated markers, while comparing follow-up to baseline measurements helped discover biomarkers and pathways involved in disease progression. The patient groups were well-matched at baseline, while eGFR naturally decreased over time in the PF group. The similarity of patient characteristics at baseline was confirmed by the PCA of the proteomic data, which showed almost complete overlap between the PBL and SBL groups. As there are very minor differences at the early stage before progression status is known, it becomes challenging to find markers of prognostic value. Despite the high similarity, a few differentially expressed proteins, such as *LALBA*, *LRRC4B*, and *HMGB1* (increased in PBL), and *FBLN2*, *QPCT*, and *EFNA1* (decreased in PBL), might serve as early prognostic biomarkers of progressive CKD. Some of these DEPs have established roles in kidney function and disease. For instance, HMGB1 is a pro-inflammatory protein involved in kidney injury and fibrosis [18] that has been proposed as target for the treatment of renal diseases [19]. Whereas circulating EFNA1 proteins have been associated with kidney failure risk among adults with moderate to severe CKD [20].

Dysregulation of pathways related to infection, bacterial invasion, and inflammation was particularly evident in the SBL and PBL comparisons. This dysregulation may result from heightened inflammatory processes, which overlap with infection pathways, such as the complement pathway. Complement dysregulation between progressors and non-progressors has also been implicated in a study based on the Salford kidney study cohort on 414 patients with a broad range of kidney disease aetiologies, including HN [12]. Our findings could also indicate low-level infection or an altered microbiota balance. CKD disrupts the symbiotic relationship between host organs and the gut microbiota, contributing to disease progression [21], and CKD-induced dysbiosis results in increased levels of microbiota-derived metabolites, such as indoxyl sulfate, which predicts a decline in renal function [22]. Furthermore, CKD patients often exhibit a leaky gut, allowing microbiota-derived products such as lipopolysaccharides to enter the systemic circulation and induce systemic inflammation [23–25]. Low-level endotoxin can activate inflammatory pathways in human blood vessels, exacerbating CKD-related complications [26].

An intriguing finding was the altered expression of PD-L2 (Programmed cell death 1 ligand 2), which was the most abundant protein in the PF/PBL and PF/SF groups. PD-L2 is an immune checkpoint receptor ligand that negatively regulates the adaptive immune response by interacting with PD-1 on various immune cells, including T lymphocytes [27, 28]. It is expressed on antigen-presenting cells [29] and certain T helper cell subsets [30], playing a crucial role on the negative regulation of CD8+ T cells activation via the PD/PD-L2 axis [31]. renal research in a PD-L2(−/−) mouse model, showed increased intrarenal leukocyte infiltrates and circulating IgG-deposited glomerular IgG, suggesting a role in immune homeostasis [32]. Decreased PD-L2 levels in CKD may lead to uncontrolled inflammation, contributing to early and advanced disease progression. As it is expressed in renal tubular cells and its abundance declines in early CKD, PD-L2 could serve as an early marker of tubular injury. The enrichment of pathways related to infection, inflammation, and immunity in advanced disease groups, further supports its potential as a key modulator of immune-driven renal deterioration [20, 33], particularly in the context of bacterial or viral infections. Moreover, PD-L2’s role in immune regulation highlights its promise as both a prognostic marker and a therapeutic target for modulating immune responses in HN and CKD.

Previous proteomics studies on microdissected glomeruli support our observations. Analysis of kidney biopsies in hypertensive nephropathy revealed disruptions in metabolic, structural, and adhesion-related pathways in CKD [34], while post-transplant diabetes and T2DM exhibited dysregulated cell adhesion and immune responses [35]. Together, these findings suggest that the pathophysiology of HN may stem from the combined effects of immune activation, metabolic stress, and impaired cellular adhesion. The shared dysregulation of these interconnected pathways highlights the importance of a comprehensive approach to both diagnosis and therapy.

Serum proteomics offers a minimally invasive approach to characterise HN by analysing serum proteins and holds the potential to provide a better understanding of processes contributing to disease progression. In HN and CKD there is a need for improved assessment and prediction of progression beyond traditional measures like eGFR [36]. Our observations regarding the involvement of the complement system in CKD are not new [37], and has been shown to be involved in non-autoimmune disorders such as diabetic nephropathy [38], and in development of hypertension [39, 40]. The C5 complement inhibitor eculizumab has shown promise in atypical haemolytic uraemic syndrome, and might have utilisation in other forms of CKD [41]. Proteomics could potentially detect subtle molecular changes before significant declines in eGFR occur, allowing for earlier intervention. Despite its potential, proteomics faces challenges due to the complexity of the proteome and technical limitations. High-abundance proteins can overshadow low-abundance ones that might be important in disease mechanisms. Some limitations in the study should be acknowledged. TMT-labelled experiments are susceptible to batch effects, which can impact the consistency and comparability of results [42]. However, any batch effects were corrected in our study. Also, many of the DEPs did not reach statistical significance after multiple testing correction, reinforcing that these proteins should not be considered definitive biomarkers but rather as candidates for further validation. Additionally, serum proteomics faces inherent challenges, such as high-abundance proteins overshadowing low-abundance ones. Plasma might be more suitable than serum for proteomics due to the clot formation involved in serum generation. This clotting process can lead to the degradation and modification of serum proteins and the release of proteins from platelets, leukocytes, and blood clots [43, 44]. Also, the study included a relatively small sample size (41 participants), and a subset of the patients did not have biopsy-proven HN, but HN was diagnosed by exclusion.

These findings underscore the biological importance of the identified proteins and their collective influence on overlapping pathways, rather than pointing to any single protein as a definitive biomarker. Our data show that coordinated disruption of inflammatory, metabolic, and adhesion pathways may drive the progression of HN. This systems-level view enhances our understanding of HN progression and identify potential molecular targets for early diagnosis and intervention. Validating these results in larger cohorts will be critical for translating this knowledge into practical clinical applications.

## Supplementary Information

Additional file 1: Figure S1. Batch correction and missing values. A) Hierarchical clustering of non-imputed or batch-corrected samples displays missing and non-missing values. The samples were clustered according to their respective batches. B) Hierarchical clustering following imputation. Missing values were considered missing because of biological differences rather than batch effects. C) Uniform manifold approximation and projection analysis before and D) after batch correction.

Additional file 2: Figure S2. Complete eGFR curves for the patients. Plots displaying the stable and progressor patient groups with eGFR values for the available study period. The time points for the follow-up proteomic measurements are indicated in red. The baseline measurement is at the start of the curves. The eGFR values for both groups were fitted with a linear model and calculated via the CKD-EPI formula. Patients who received renal replacement therapy are highlighted in black.

Additional file 3: Figure S3. PCA plots annotated with clinical data show no separation between groupsPrincipal component analysis (PCA) plots of the protein expression data, annotated with clinical variables including: **A** Gender, **B** Smoking, **C** Alcohol Consumption, **D** Diabetes, **E** Myocardial infarction,**F** Angina, **G** CCF, **H** ACE/ARB treatment, **I** Statin use, **J** Death, and **K** RRT. Each plot represents the distribution of samples based on the specified variable, with no clear separation between groups observed across the principal components. Ellipses represent 95% confidence intervals.

Additional file 4.

Additional file 5.

Additional file 6.

Additional file 7.

Additional file 8.

## Data Availability

The data has been submitted to proteome exchange, please use the information provided below to access the data. Scripts for data analysis is available on request. Project Name: Longitudinal Serum Proteomics in Hypertensive Kidney Disease Progression Project accession: PXD055241 Project DOI: 10.6019/PXD055241.

## Abbreviations

HN: Hypertensive nephropathy.
TMT: Tandem mass tag
LC‒MS: Liquid chromatography-tandem mass-spectrometry
CKD: Chronic kidney disease
eGFR: Estimated glomerular filtration rate
TFA: Trifluoroacetic acid
ACN: Acetonitrile
FA: Formic acid
PBL: Progressive baseline
PF: Progressive follow-up
SBL: Stable baseline
SF: Stable follow-up
UMAP: Uniform manifold approximation and projection
PCA: Principal component analysis
HPA: Human protein atlas
SD: Standard deviation
DEP: Differentially expressed proteins
BMI: Body mass index
BP: Blood pressure
log_2_FC: Log_2_ Fold change
