# Supplementary figures and images for: Longitudinal serum proteomics identifies inflammatory and metabolic pathways in hypertensive nephrosclerosis progression
Source: Clin Proteomics. 2025 May 5;22:17. doi: 10.1186/s12014-025-09537-5 (PMC12054191; doi:10.1186/s12014-025-09537-5)

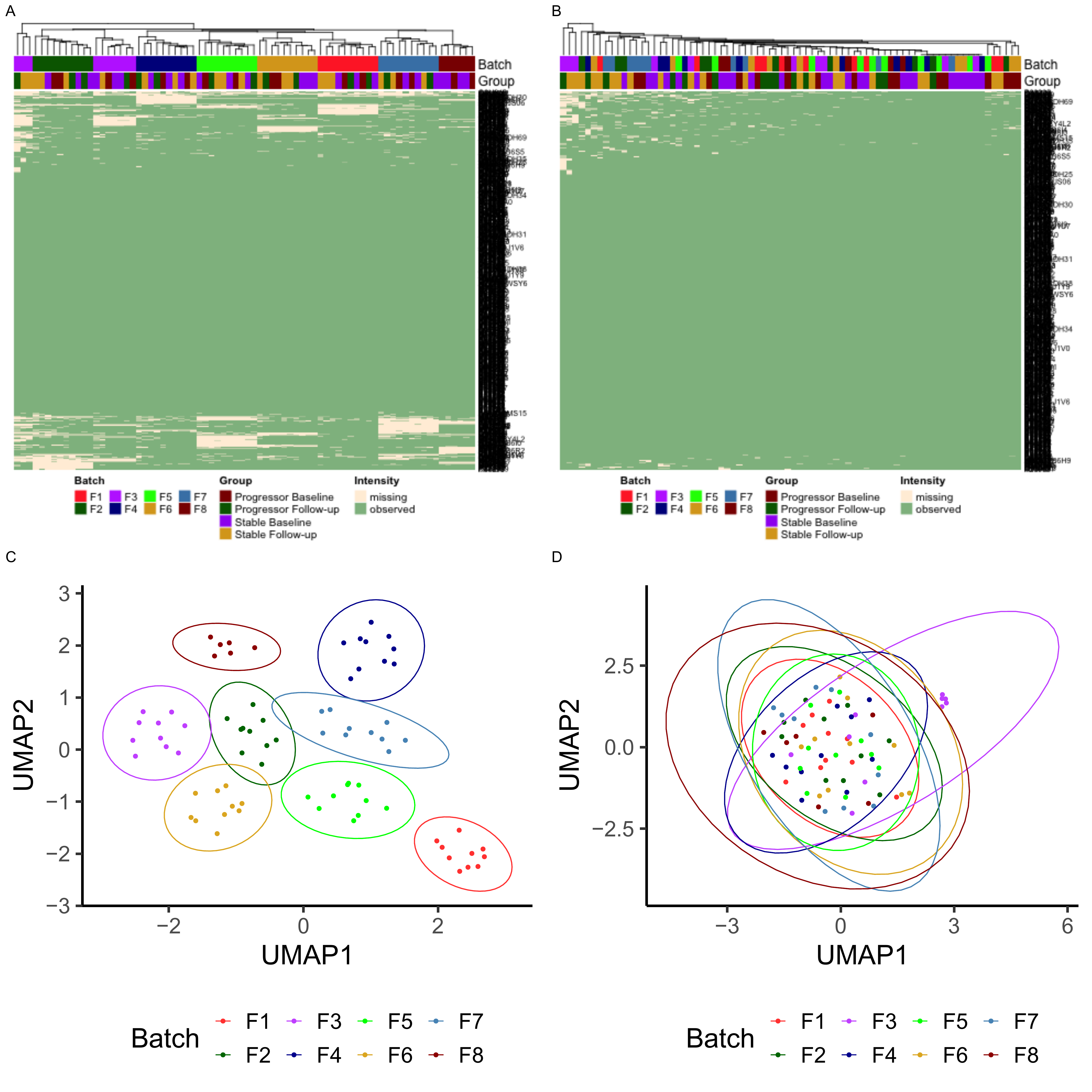

Supplement: Supplementary file 1 — Additional file 1: Figure S1. Batch correction and missing values. A) Hierarchical clustering of non-imputed or batch-corrected samples displays missing and non-missing values. The samples were clustered according to their respective batches. B) Hierarchical clustering following imputation. Missing values were considered missing because of biological differences rather than batch effects. C) Uniform manifold approximation and projection analysis before and D) after batch correction. [file 12014_2025_9537_MOESM1_ESM.jpeg]

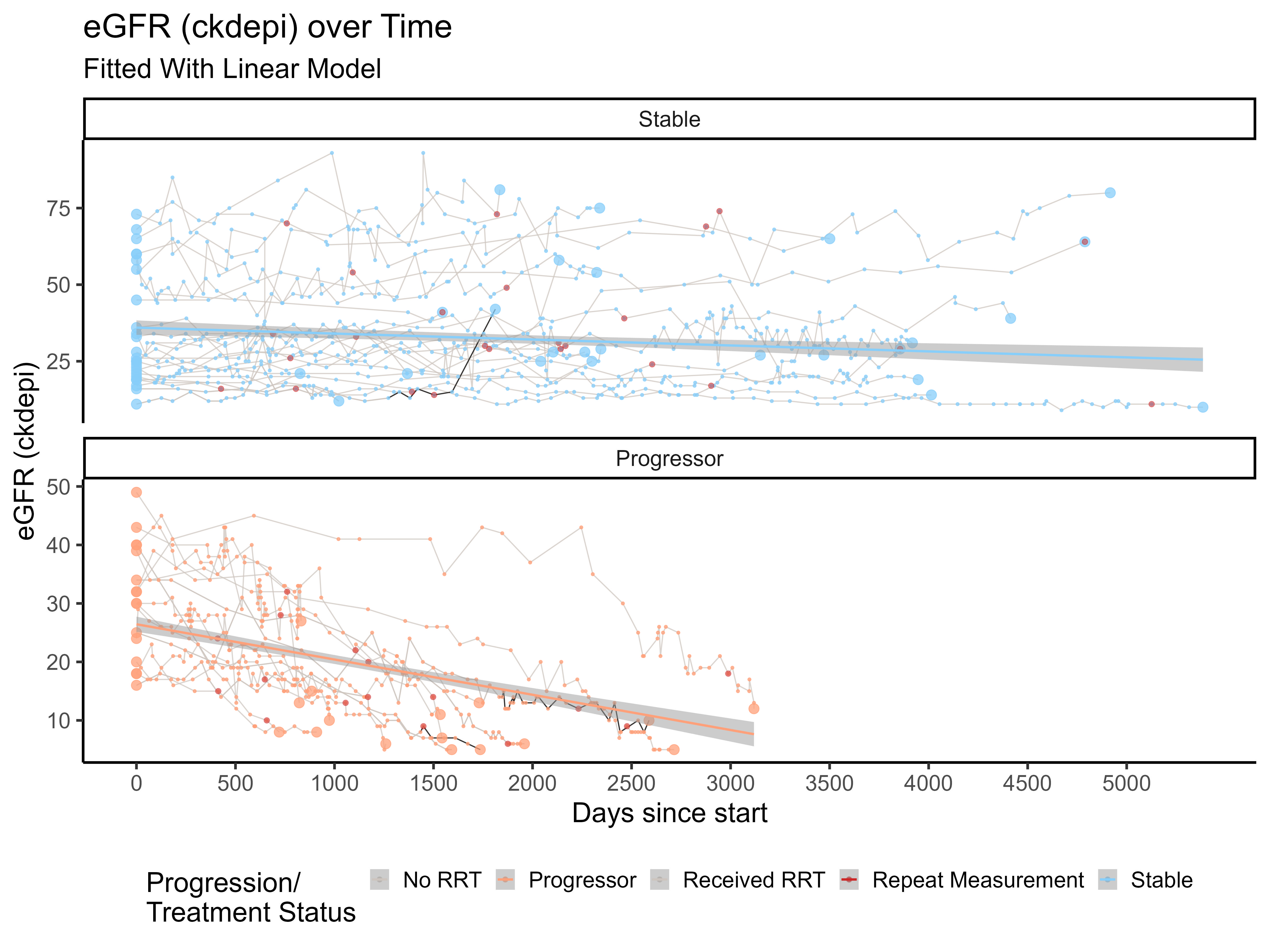

Supplement: Supplementary file 2 — Additional file 2: Figure S2. Complete eGFR curves for the patients. Plots displaying the stable and progressor patient groups with eGFR values for the available study period. The time points for the follow-up proteomic measurements are indicated in red. The baseline measurement is at the start of the curves. The eGFR values for both groups were fitted with a linear model and calculated via the CKD-EPI formula. Patients who received renal replacement therapy are highlighted in black. [file 12014_2025_9537_MOESM2_ESM.jpeg]

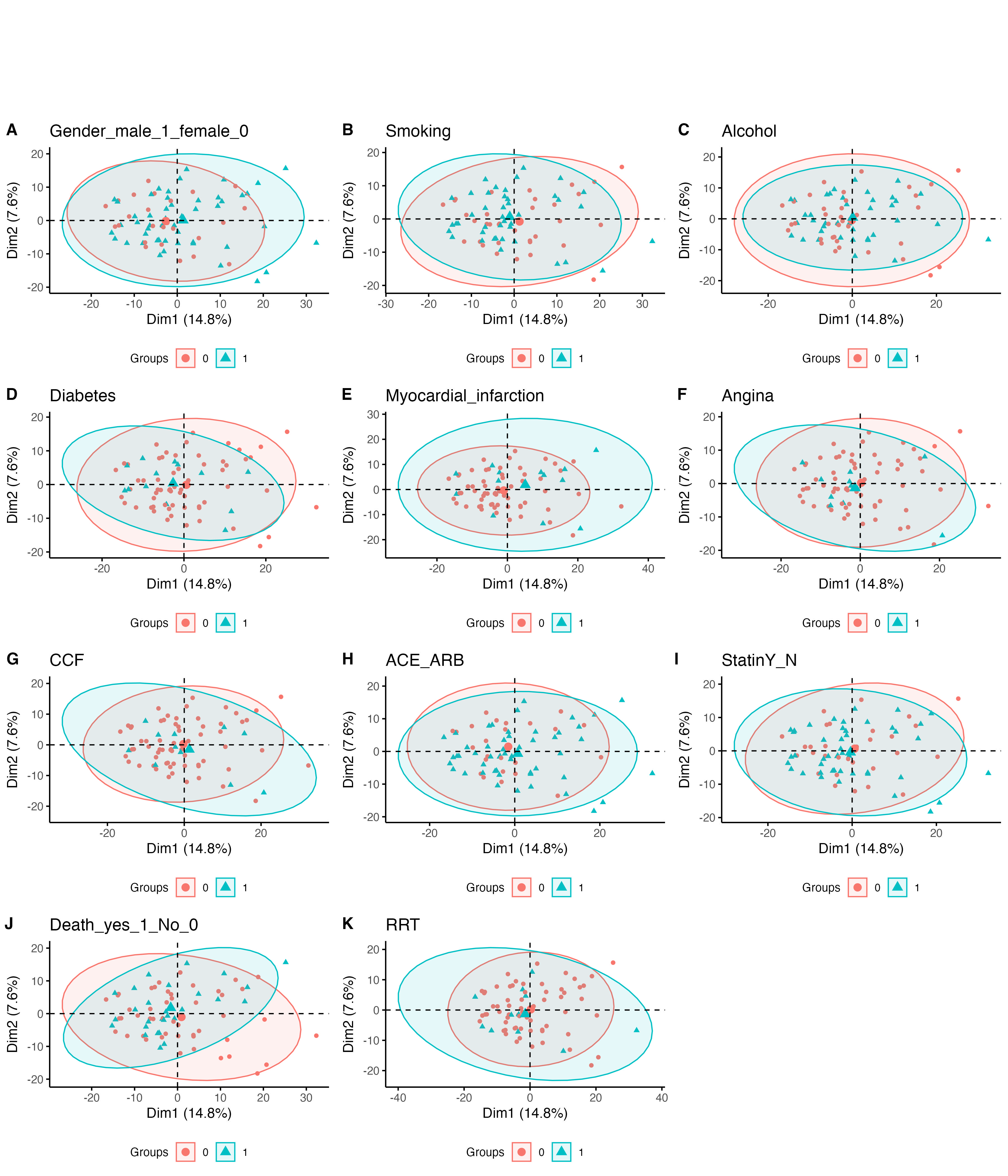

Supplement: Supplementary file 3 — Additional file 3: Figure S3. PCA plots annotated with clinical data show no separation between groupsPrincipal component analysis (PCA) plots of the protein expression data, annotated with clinical variables including: A Gender, B Smoking, C Alcohol Consumption, D Diabetes, E Myocardial infarction,F Angina, G CCF, H ACE/ARB treatment, I Statin use, J Death, and K RRT. Each plot represents the distribution of samples based on the specified variable, with no clear separation between groups observed across the principal components. Ellipses represent 95% confidence intervals. [file 12014_2025_9537_MOESM3_ESM.jpeg]
